# In silico repurposing of antipsychotic drugs for Alzheimer’s disease

**DOI:** 10.1186/s12868-017-0394-8

**Published:** 2017-10-27

**Authors:** Shivani Kumar, Suman Chowdhury, Suresh Kumar

**Affiliations:** University School of Biotechnology, GGS Indraprastha University, Sector-16C, Dwarka, New Delhi 110075 India

**Keywords:** Drug repurposing, Alzheimer’s disease, Antipsychotic drugs, Acetylcholinesterase, Butyrylcholinesterase, Beta-secretase cleavage enzyme, Monoamine oxidase, *N*-Methyl-d-aspartate, Molecular docking, Schrodinger

## Abstract

**Background:**

Alzheimer’s disease (AD) is the most prevalent form of dementia and represents one of the highest unmet requirements in medicine today. There is shortage of novel molecules entering into market because of poor pharmacokinetic properties and safety issues. Drug repurposing offers an opportunity to reinvigorate the slowing drug discovery process by finding new uses for existing drugs. The major advantage of the drug repurposing approach is that the safety issues are already investigated in the clinical trials and the drugs are commercially available in the marketplace. As this approach provides an effective solution to hasten the process of providing new alternative drugs for AD, the current study shows the molecular interaction of already known antipsychotic drugs with the different protein targets implicated in AD using in silico studies.

**Result:**

A computational method based on ligand–protein interaction was adopted in present study to explore potential antipsychotic drugs for the treatment of AD. The screening of approximately 150 antipsychotic drugs was performed on five major protein targets (AChE, BuChE, BACE 1, MAO and NMDA) by molecular docking. In this study, for each protein target, the best drug was identified on the basis of dock score and glide energy. The top hits were then compared with the already known inhibitor of the respective proteins. Some of the drugs showed relatively better docking score and binding energies as compared to the already known inhibitors of the respective targets. Molecular descriptors like molecular weight, number of hydrogen bond donors, acceptors, predicted octanol/water partition coefficient and percentage human oral absorption were also analysed to determine the in silico ADME properties of these drugs and all were found in the acceptable range and follows Lipinski’s rule.

**Conclusion:**

The present study have led to unravel the potential of leading antipsychotic drugs such as pimozide, bromperidol, melperone, anisoperidone, benperidol and anisopirol against multiple targets associated with AD. Benperidol was found to be the best candidate drug interacting with different target proteins involved in AD.

## Background

Alzheimer’s disease (AD) is the most prevalent form of dementia associated with progressive cognitive deterioration, behavioural and neuropsychiatric symptoms [1, 2]. There are approximately 35 million people worldwide and 3.7 million in India suffering from AD. About one in ten adults over 65 and almost 50% of the people above 85 years of age develops AD [3]. Currently, commercially available drugs used for symptomatic treatment of AD such as neostigmine, physostigmine, rivastigmine, donepezil, tacrine and memantine show side effects such as gastrointestinal disturbances, muscle aches, vomiting, heartburn, headache, loss of appetite, diarrhoea, loss of balance, hepatoxicity and shorter half-life [4]. In view of these shortcomings there is continues search for new drugs with lesser side effects. In the last few years less than 25 drugs are in phase II and III clinical trials for AD, whereas more than 1700 are there for cancer therapies [5].

Drug repurposing is the process of evaluating the applicability of already known drug for their new therapeutic role. Drug repurposing has already been practiced in many therapies such as cancer, cardiovascular disease, stress incontinence, irritable bowel syndrome, erectile dysfunction, obesity, smoking cessation, psychosis, attention deficit disorder and Parkinson’s disease [6]. With already established drug compounds, the advantages are that it save time and cost on preliminary clinical trials such as chemical optimization, in vitro and in vivo screening, toxicology studies, bulk manufacturing and formulation development [7]. Whereas, a new drug candidate takes billion of dollars and at least 15 years to come in the market [8]. In fact, one of the establish drug for AD, Galanthamine, an acetylcholinesterase (AChE) inhibitor was earlier used for Poliomyelitis in Eastern Europe and then repurposed for use in AD same as Lundbeck repurposed memantine for therapeutic use in AD as Ebixa^®^ [9, 10]. Other examples include citalopram, desvenlafaxine, and fluoxetine (Selective Serotonin Reuptake Inhibitors), levetiracetam (antiepileptic drug), perindopril, nilvadipine, carvedilol (antihypertensive drugs), liraglutide, lixisenatide, metformin, exenatide (anti-diabetes drugs) all have shown to be significant in AD [11].

Various neuropathological symptoms of AD include deposition of senile neurotic plaques, loss of cholinergic neurons and formation neurofibrillary tangles in the central nervous system (CNS) [12]. There are many hypotheses to explain the cause of AD, such as cholinergic hypothesis, β-Amyloid hypothesis, glutamatergic and excitotoxic hypothesis, oxidative hypothesis and tau hypothesis [13].

### Cholinergic hypothesis

Acetylcholine (ACh), one of the most important neurotransmitter found in CNS is hydrolyzed by cholinesterase i.e., acetylcholinesterase (AChE) and butyrylcholinesterase (BuChE) enzymes. The cognitive impairment is mainly due to loss of neurotransmitter ACh caused by reduced activity of choline acetyltransferase (ChAT), an enzyme evolved in synthesis of ACh. In view of this, the main emphasis is on anticholinergic drugs, which can inhibit both the enzymes and up-regulate the level of ACh in the CNS [14]. Studies have shown that in patients of AD, BuChE activity increases from 40 to 90%, whereas AChE activity remains the same or declines [15]. Evidences have shown that AChE and BuChE both plays an important role in accelerated pro-aggregation of β-Amyloid (Aβ) fibrils formation [16]. Cholinesterase inhibitors, which bind to the peripheral anionic site (PAS) of AChE have found to inhibit AChE-induced aggregation of Aβ fibrils [17].

### β-Amyloid cascade hypothesis

According to this hypothesis there is an overproduction and aggregation of Aβ peptide, leading to formation of neuritic plaques in CNS [18]. Enzymes like Beta-secretase cleavage enzyme (BACE 1) and Gamma-secretase (γ secretase) are responsible for the formation of these peptides by the proteolytic cleavage of amyloid precursor protein (APP), producing two variables peptide i.e. Aβ_40_ and Aβ_42_. Both Aβ_40_ and Aβ_42_ peptide aggregation leads to neurotoxicity and neural cell death [19, 20]. Aβ_1–42_ polypeptide is more prone to form insoluble and toxic aggregates as compared to Aβ_1–40_ polypeptide [21]. BACE 1 enzyme is one of the important drug targets for the development of anti-Alzheimer’s drug because of its role in β-amyloid cascade. Inhibition of BACE 1 enzyme at the beginning of APP processing would prevent the formation of insoluble toxic Aβ aggregates that is responsible for neurodegeneration in AD.

### Oxidative hypothesis

According to the hypothesis, the reactive oxygen species (ROS) formation increases within the mitochondria under stressful conditions and in aging, with no efficient antioxidant system it leads to the risk of developing AD. The brain of AD patients shows a significant extent of oxidative damage. ROS activates BACE 1 and γ-secretase enzymes to increase Aβ production and abnormal accumulation of Aβ fibrils in the brain of AD patients from APP. Aβ and APP may themselves also directly induce the production of ROS [22, 23]. An enzyme namely monoamine oxidase (MAO) is proposed to be involved in AD due to elevated production of ROS. MAO has two subtypes i.e. MAO A and MAO B responsible for the catalysis the deamination of xenobiotic and biogenic amines, like norepinephrine, dopamine and serotonin [24, 25]. Studies have also shown that there is an increased level of neurotransmitters in the CNS, when MAO is being inhibited [26, 27].

### Glutamatergic and excitotoxic hypothesis

Glutamate is the major excitatory neurotransmitter, involved in synaptic plasticity, memory and learning in the cortical and hippocampal region of CNS [28, 29]. Dysfunction in the glutamatergic system has been linked with increase in oxidative stress associated with the Aβ peptide, the pathophysiological processes underlying AD. Glutamate synthase being sensitive to oxidative stress leads to decrease in its activity and resulting in increased glutamate levels [30]. The *N*-methyl-d-aspartate (NMDA) receptor is a member of the family of ionotropic glutamate receptors. Its over-activation due to excessive glutamate causes continuous influx of calcium ions (Ca^2+^) into the nerve cells, ultimately leading to cell death [31]. Previous studies suggests that NMDA receptors are constantly hyper-activated, generating a form of ‘slow excitotoxicity’ at post synaptic neurons, producing a gradual neurodegenerative effect in AD patients [32]. Memantine, one of the United States Food and Drug Administration (FDA) approved drug for AD is a NMDA receptor antagonist. Therefore, NMDA receptor antagonists could be therapeutically beneficial in AD.

Most of the patients having dementia suffer from behavioural and psychological symptoms of dementia (BPSD) which includes depression, aggression, apathy, delusions, agitation, euphoria, hallucinations and sleep disturbances. Antipsychotics or neuroleptics are the class of drugs that are used for the management of psychosis mainly bipolar disorder, schizophrenia and for some non-psychotic disorders including depression and anxiety [33]. There are two major groups of antipsychotic drugs namely typical and atypical antipsychotic. Typical or first generation antipsychotics inhibit dopaminergic neurotransmission whereas the atypical or second generation antipsychotics are dopamine D2 and serotonin (5-HT, 5-hydroxytryptamine) receptor antagonist [34]. Antipsychotic drugs have shown to be interacting with major 5-HT receptors such as 5-HT_1a_, 5-HT_2a_, 5-HT_2c_, 5-HT_3_, 5-HT_6_, and 5-HT_7_ receptors [35]. Antipsychotic drugs like aripiprazole, haloperidol, ziprasidone, clozapine, quetiapine are already in use for treating BPSD in AD patients. Lecozotan, a 5-HT_1A_ receptor antagonist has cleared phase III clinical trial as cognitive enhancers in patients with AD [33, 36].

Due to the complex nature of AD, the conventional “one molecule, one target” approach may not offer the best pathway for this complex disease. Therefore, the multi-target directed strategy is much needed for disease modifying therapeutics. In view of this, present study demonstrates the interactions of screened antipsychotic drugs with the multiple targets of AD, using molecular docking and in silico ADME studies.

## Methodology

### Protein preparation

**Fig. 1:**
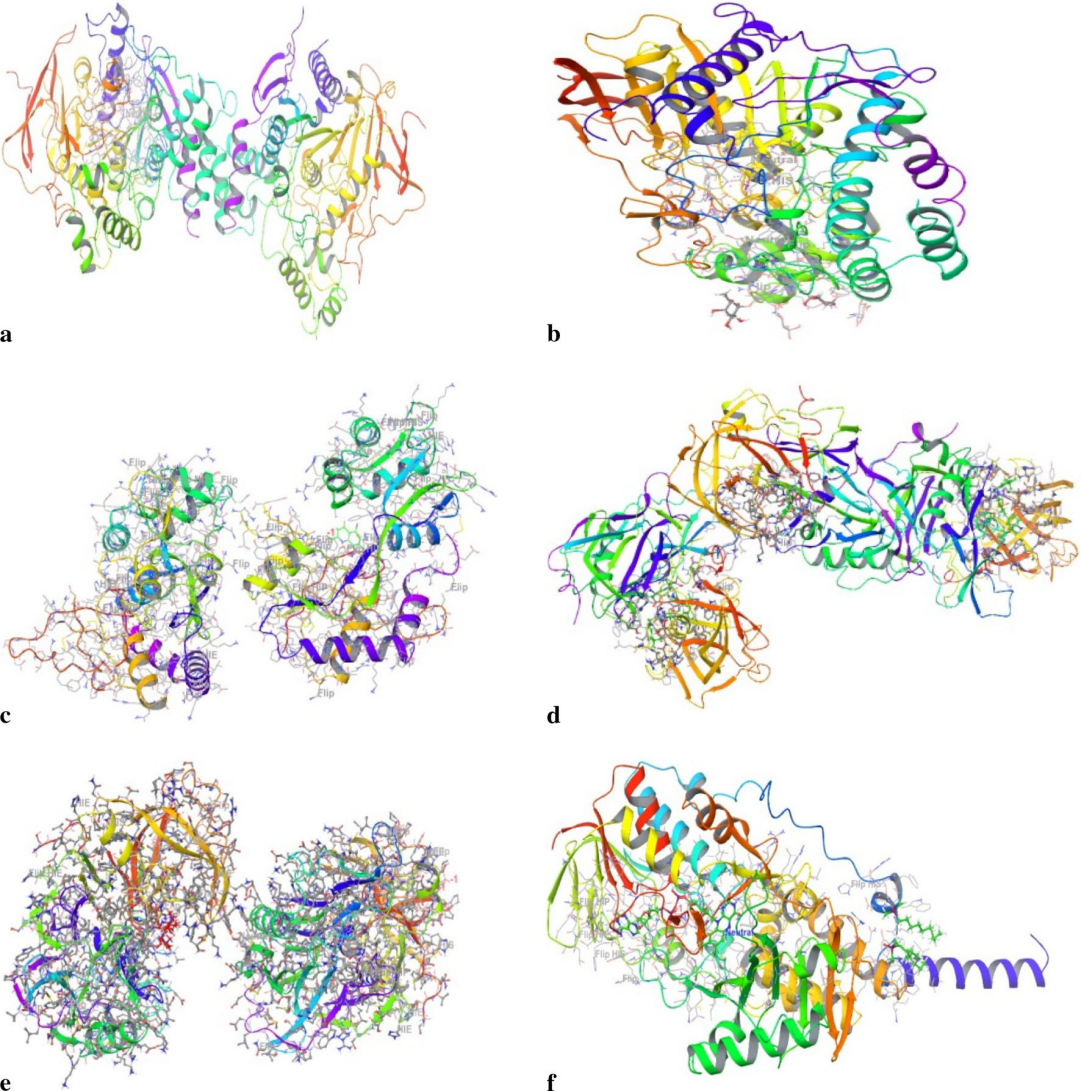
The crystallographic structure of the prepared proteins used for docking studies (**a** 4EY6; **b** 1P0M; **c** 1PBQ; **d** 4D8C; **e** 3L5E; **f** 2Z5X)

X-Ray crystallographic structure of all the target proteins i.e. AChE (4EY6), BuChE (1POM), MAO A (2Z5X), BACE 1 (4D8C, 3L5E) and NMDA (1PBQ) structure was downloaded from Protein Data Bank (PDB). Before performing molecular docking, all the protein structures were prepared using ‘Protein Preparation Wizard’ workflow in Schrodinger suite. This involved addition of hydrogen atoms to the protein, assignment of bond orders, and deletion of unnecessary water molecules. The important water molecules found to be interacting with the active site residues of the enzyme were retained. In case of 4EY6, seven water molecules [37]; for 1POM, two water molecules [38]; two water molecule in 4D8C, one in 3L5E [39] and one in 1PBQ [40]. No water molecules were retained in 2Z5X [41]. Side chains were added, disulphide bonds were formed, missing atoms were added and the partial charges were assigned. Energy minimization was done using OPLS_2005 (Optimized Potentials for Liquid Simulations) force field (Fig. 1). As all the downloaded proteins were co-crystallised structures, the ligand binding site were used so as to define the active site of the protein. Receptor grid generation workflow was used to define a grid (box) around the ligand, to keep all the functional residues in the grid [42].

### Ligand preparation

Based on literature review, approximately 150 antipsychotic drugs were selected and their 3D structures were downloaded from Pubchem. Using Ligprep, pre-processing of the ligands were done, which includes formation of tautomers and ionization states at pH 7.0 ± 2.0 using Epik, hydrogen atoms were added, charged groups were neutralised and geometry of the ligands were optimised [43].

### Molecular docking

Using glide (grid based ligand docking with energetics) algorithm, extra precision docking was performed with the prepared protein and the ligands. Structures of ligands were kept flexible to generate different conformations. OPLS force fields were used to perform these calculations [44]. All the Glide docking runs were performed on Intel^®^ Core™ i7-3770 CPU @ 3.40 GHz of HP origin, with 4 GB RAM, Windows 8Pro operating system. All the results were analyzed in XP visualizer.

### Pharmacokinetic parameters

QikProp in Schrodinger suite was used to calculate the physiochemical properties of the drugs like molecular weight of the compounds (MW), Predicted octanol/water partition coefficient (QPlogPo/w), the number of hydrogen bond donors (donorHB), acceptors (accptHB) and percentage human oral absorption, violations of Lipinski’s rule of five were also analyzed [45].

## Results

The main focus of present study is to identify antipsychotic drugs as repurposed drugs for the treatment of AD. Approximately, 150 antipsychotic drugs were screened using molecular docking. Out of the screened drugs, sulmepride, promazine hydrochloride, bromperidol, anisopirol, melperone, pimozide, benperidol, azabuperone and anisoperidone significantly interacted with selected protein targets of AD. The docking score was observed in the range of − 10.927 to − 14.969 in case AChE (4EY6), − 6.663 to − 8.13 in case of BuChE (1P0M), − 5.26 to − 8.91 in MAO A (2Z5X), − 5.907 to − 8.513 in BACE 1 (3L5E), − 3.649 to − 9.076 in BACE 1 (4D8C) and − 4.711 to − 6.903 in NMDA (1PBQ) (Table 1). Among these antipsychotic drugs, the best hit for each target was selected on the basis of docking score and binding energy. There binding modes and the molecular interactions were also compared with potent known drugs/inhibitors in the crystal structures of the target protein. The binding energies in terms of glide energy, energy contributed due to electrostatic bonding (ΔG_ecol_), Van der Waals (ΔG_edw_), hydrogen bonding (xphbond), interacting residues (both hydrophilic as well as hydrophobic) and their bond length of the best predicted drug with each protein target are presented in Table 2.

**Table 1:** Dock score of the top nine interacting drugs with multiple target proteins

Name of the ligand	Docking score					
	AChE (4EY6)	BuChE (1P0M)	MAO A (2Z5X)	BACE 1 (3L5E)	BACE 1 (4D8C)	NMDA (1PBQ)
Positive control	Donepezil − 11.02	Rivastigmine − 3.123	Marplan − 8.354	LY2886721 − 6.123	LY2886721 − 6.80	Memantine − 4.029
Sulmepride	− 11.508	–	–	− 7.246	− 3.649	− 4.831
Promazine hydrochloride	− 11.074	–	− 7.978	− 5.907	− 6.784	− 4.711
Bromperidol	− 10.927	− 8.111	− 5.52	− 6.576	− 7.486	− 5.247
Anisopirol	− 11.001	− 7.977	− 5.26	− 6.083	− 8.169	− 6.903
Melperone	− 11.423	− 6.663	− 8.91	− 7.249	− 6.524	− 6.067
Pimozide	− 14.969	− 8.13	–	− 8.386	− 8.968	− 6.854
Benperidol	− 14.425	− 7.163	− 6.598	− 6.742	− 9.076	− 6.778
Azabuperone	− 11.882	− 7.938	− 5.861	− 7.584	− 4.167	− 6.51
Anisoperidone	–	− 7.989	− 6.324	− 8.513	− 7.11	− 5.196

**Table 2:** Molecular interaction of drug targets with putative* and known inhibitor^#^

Target	Drug name	Glide energy (kcal/mol)	ΔG_edw_ (kcal/mol)	ΔG_ecol_ (kcal/mol)	Xphbond (kcal/mol)	Interacting residues/molecule	Bond length (Ǻ)
AChE	Pimozide*	− 50.362	− 39.030	− 11.332	0.700	Tyr124(H bond)	2.05
						Trp286 (π–π stacking)	3.72
						Ser293 (H bond)	2.64
						Phe295 (H bond)	1.96
						Phe338 (π–π stacking)	5.18
						Tyr341 (π-cation)	4.81
	Donepezil^#^	− 27.944	− 25.03	− 2.911	0.000	Trp86 (π–π stacking)	3.98587
						Trp286 (π–π stacking)	4.2134
BuChE	Bromperidol*	− 42.936	− 38.35	− 4.584	− 0.7	Pro285 (H bond)	2.04008
						Phe329 (π–π stacking)	5.42016
	Rivastigmine^#^	− 35.510	− 30.006	− 4.850	0	Tyr332 (π–π stacking)	3.13
MAO A	Melperone*	− 29.292	− 25.99	− 3.301	0	Phe208 (H bond)	2.06394
						Phe208 (π-cation)	4.06362
	Marplan^#^	− 36.965	− 32.950	− 4.015	− 0.689	Gln215 (H bond)	2.26
						Asn181 (H bond)	2.23
						Tyr407 (π–π stacking)	3.65
BACE 1 (3L5E)	Anisoperidone*	− 43.063	− 34.559	− 8.505	− 0.99	Trp137 (H bond)	1.9793
						Tyr259 (H bond)	2.5579
						Tyr132 (π-cation)	5.55641
						Asp93 (salt bridge)	4.41983
	LY2886721^#^	− 35.835	− 34.465	− 1.370	− 0.934	Gln134 (H bond)	2.19
						Lys168 (H bond)	2.73
						Phe169 (π–π stacking)	5.29
BACE 1 (4D8C)	Benperidol*	− 54.082	− 42.994	− 11.088	− 1.254	Thr72 (H bond)	2.09515
						Phe108 (H bond)	2.13012
						Asp32 (salt bridge)	4.14004
						Asp217 (salt bridge)	4.27946
	LY2886721^#^	− 42.84	− 32.623	− 10.224	− 2.059	Thr72 (H bond)	1.88
						Gln73 (H bond)	2.12
						Thr220 (H bond)	2.30
NMDA	Anisopirol*	− 36.533	− 34.095	− 2.437	− 1.134	Phe246 (H bond)	1.91824
						Trp223 (π–π stacking)	5.25223
	DCKA^#^	− 43.865	− 28.096	− 15.769	− 3.422	Pro124 (H bond)	2.148
						Thr126 (H bond)	1.740
						Arg131 (two H bond)	1.756
							1.822

### Validation of docking protocol

**Fig. 2:**
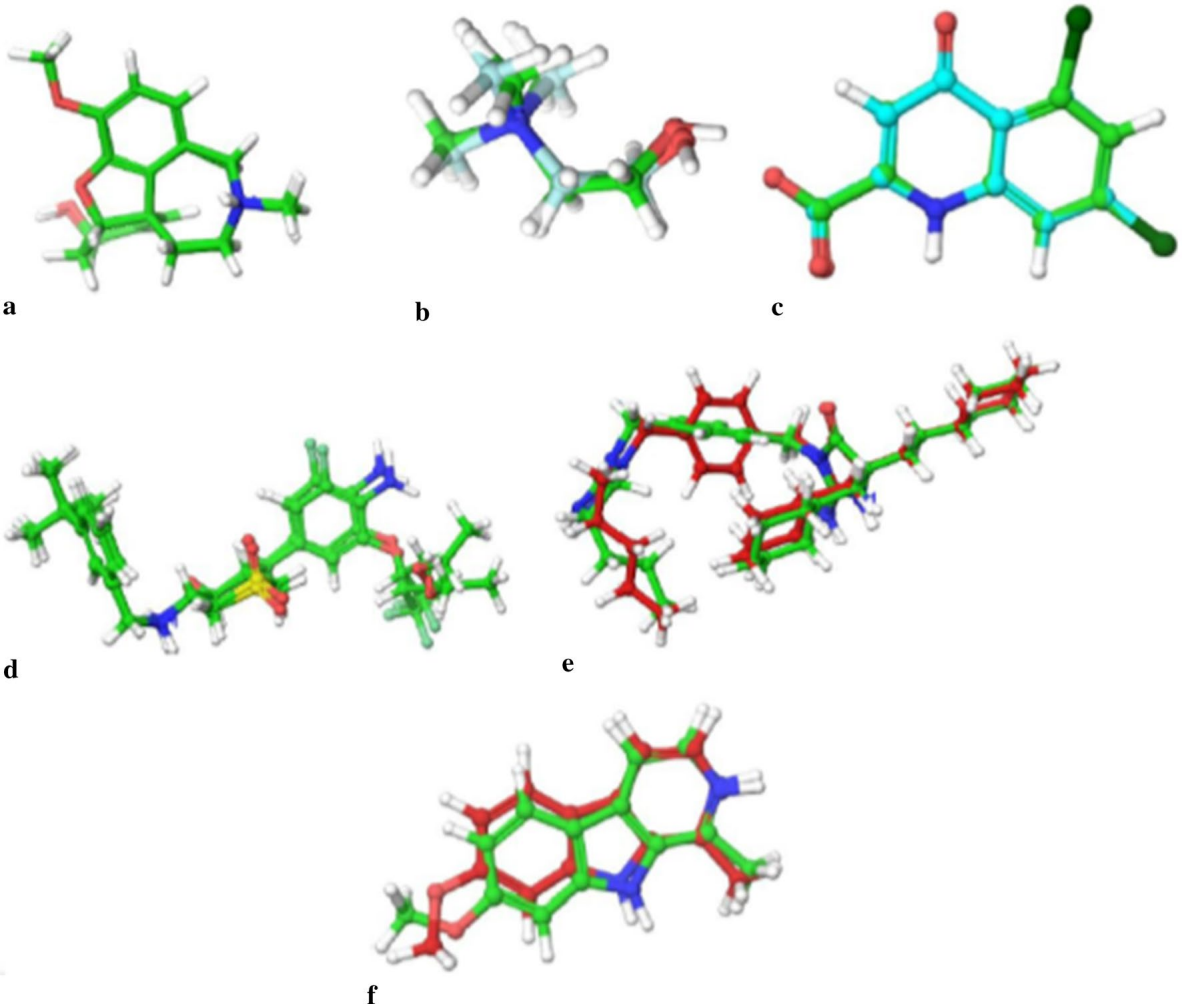
The validation of accuracy and performance of the docking protocol. **a** 4EY6 (RMSD = 0.1273 Ǻ); **b** 1P0M (RMSD = 0.9979 Ǻ); **c** 1PBQ (RMSD = 0.0974 Ǻ); **d** 4D8C (RMSD = 0.7691 Ǻ); **e** 3L5E (RMSD = 0.9404 Ǻ) **f** 2Z5X (RMSD = 0.8503 Ǻ)

For all the target proteins, the ligand in native co-crystallised structure, was extracted and was re-docked with the cavity of the respective protein molecule in order to validate the reliability, reproducibility and docking calculations. It was observed that the co-crystallised ligand was almost superimposing with the respective docked conformation of the ligands (Fig. 2). As a common rule, the scoring function is successful, if the bound ligands conformation in the experimental downloaded structure resembles the docked conformation of the respective ligand. In this study, root mean square deviation (RMSD) of all the docked complexes was in the range of 0.0974–0.9979 Å, indicating the validation of the docking protocol [46].

### Molecular interaction of the pimozide with acetylcholinesterase

**Fig. 3:**
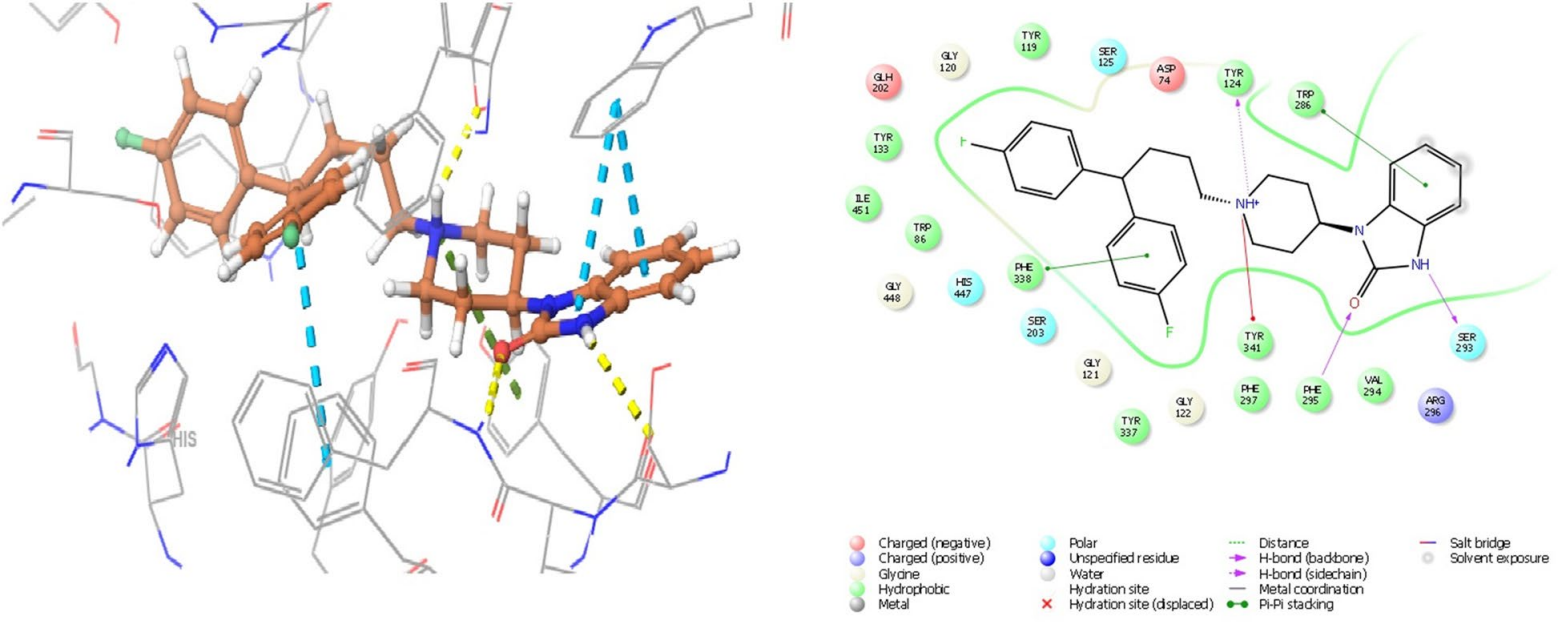
The lowest energy conformation of docking result of Pimozide with AChE (4EY6)

The active site of human AChE is 20 Å deep which has the catalytic site of human AChE (Ser 203, His447 and Glu334), acyl-binding pocket (Phe295and Phe297) at the base of the gorge, oxyanion hole (Gly120, Gly121, and Ala204), quaternary ammonium binding locus (Trp86) and lastly, PAS (Tyr72, Asp74, Tyr124, Trp286 and Tyr341) in mammals, which clusters at the entry of active site gorge [47]. In case of AChE, the best docking was observed with pimozide with dock score of − 14.969 and glide energy of − 50.362 kcal/mol and that of known inhibitor donepezil was − 11.02 and glide energy was − 27.944 kcal/mol. Pimozide forms hydrogen bonding with Tyr124, Phe295 and Ser293; pi–pi stacking with Trp286 (PAS residue), Phe338 and salt bridge with Tyr341 (PAS residue). Polar interactions were observed with catalytic residues Ser203 and His324 (Tables 1, 2 and Fig. 3).

### Molecular interaction of the bromperidol with butyrylcholinesterase

**Fig. 4:**
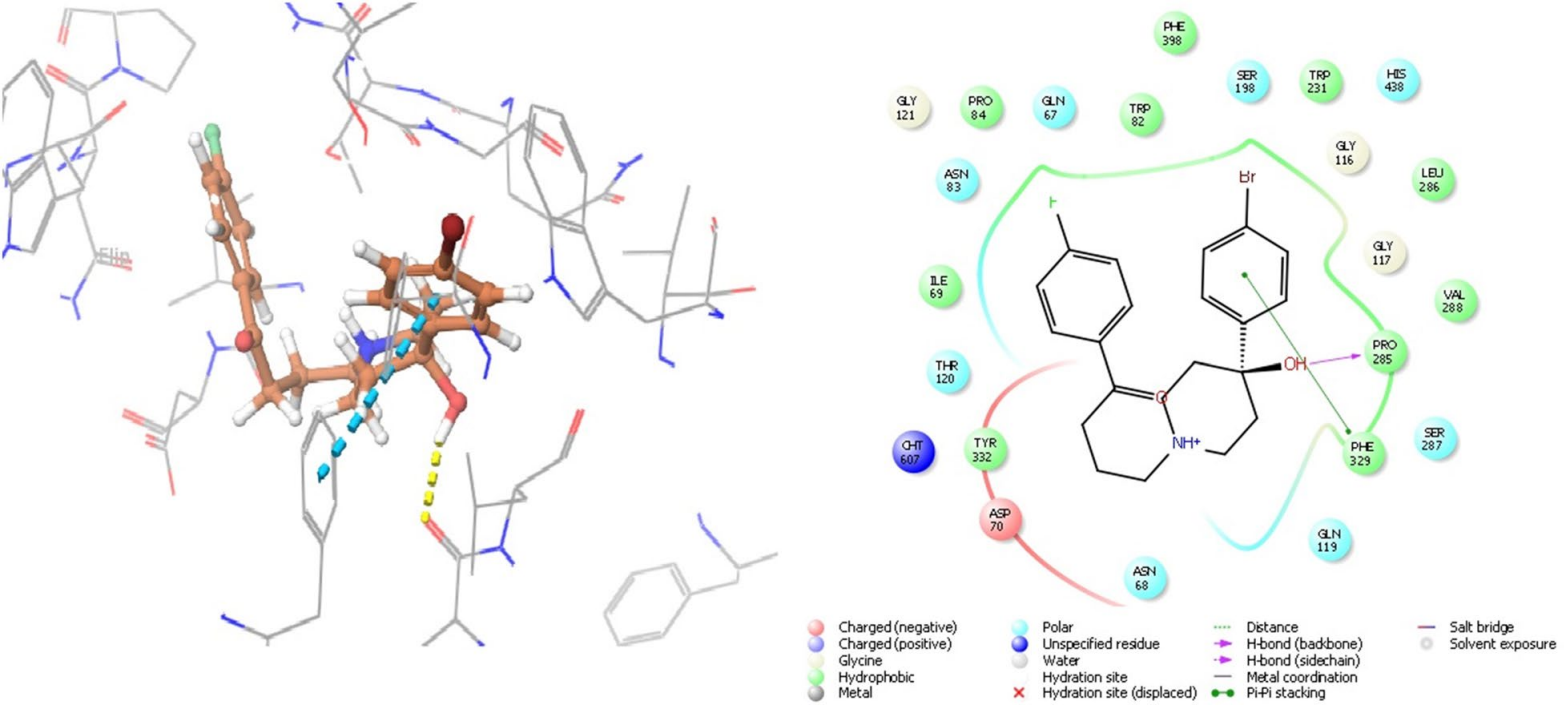
The lowest energy conformation of docking result of Bromperidol with BuChE (1P0M)

The catalytic site of human BuChE has an active site of 20 Å; its catalytic site has three residues (Ser198, His438, Glu325), choline binding site or the cation-π site (Trp82), oxyanion hole (Gly116, Gly117, Ala199), acyl binding site (hydrophobic pocket) (Leu286,Val288) and PAS (Asp70) [48]. Bromperidol showed best docking score with BuChE of − 8.111 and glide energy of − 42.936 kcal/mol and of the known inhibitor rivastigmine was − 3.123 and glide energy of − 35.510 kcal/mol. It forms hydrogen bond with Pro285, pi–pi stacking with Phe329 and polar interactions with catalytic site residues Ser198, His438 (Tables 1, 2 and Fig. 4).

### Molecular interaction of the melperone with monoamine oxidase

**Fig. 5:**
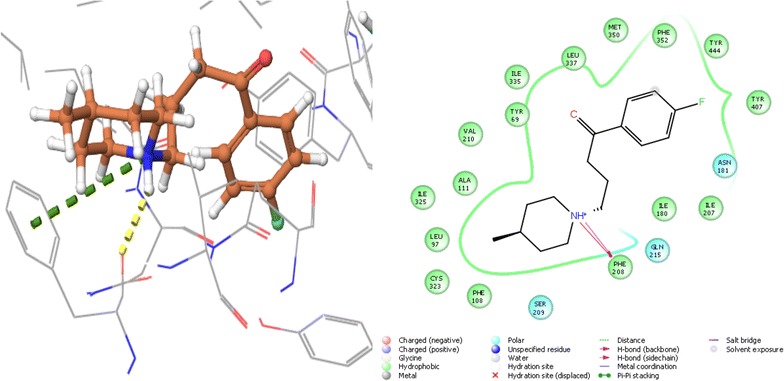
The lowest energy conformation of docking result of Melperone with MAO A (2Z5X)

MAO A has hydrophobic cavity which has a volume of ~ 400 Å. Depending upon the conformation of Phe208, MAO A can be a large single cavity or a bipartite cavity but in this case it does not work as gating residue. The enzyme has conserved active site residues which includes a pair of Tyr of the “aromatic sandwich” and a Lys hydrogen bonded to the N(5) position of the flavin i.e. Lys305 in MAO A. There are other non-conserves active site residues mainly Asn181 and Ile180 in MAO A. The major determinant in controlling the differential inhibitor and substrate specificities of these enzymes is Phe208–Ile335 in MAO A [49]. In case MAO A, the least docking score was of − 8.91 with melperone and glide energy with − 29.292 kcal/mol and in the case of known inhibitor, marplan, the docking score was − 8.777 and glide energy was − 39.947 kcal/mol. Docking results showed both hydrogen bonding and pi-cation with Phe208 (Tables 1, 2 and Fig. 5).

### Molecular interaction of the benperidol and anisoperidone with beta-secretase cleavage enzyme

**Fig. 6:**
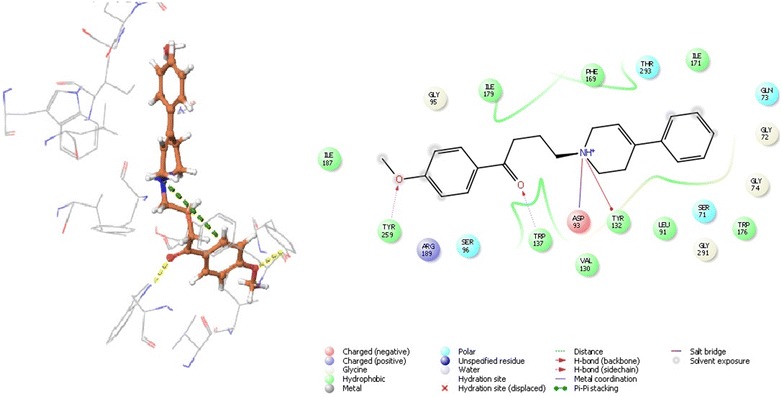
The lowest energy conformation of docking result of Anisoperidone with BACE 1 (3L5E)

**Fig. 7:**
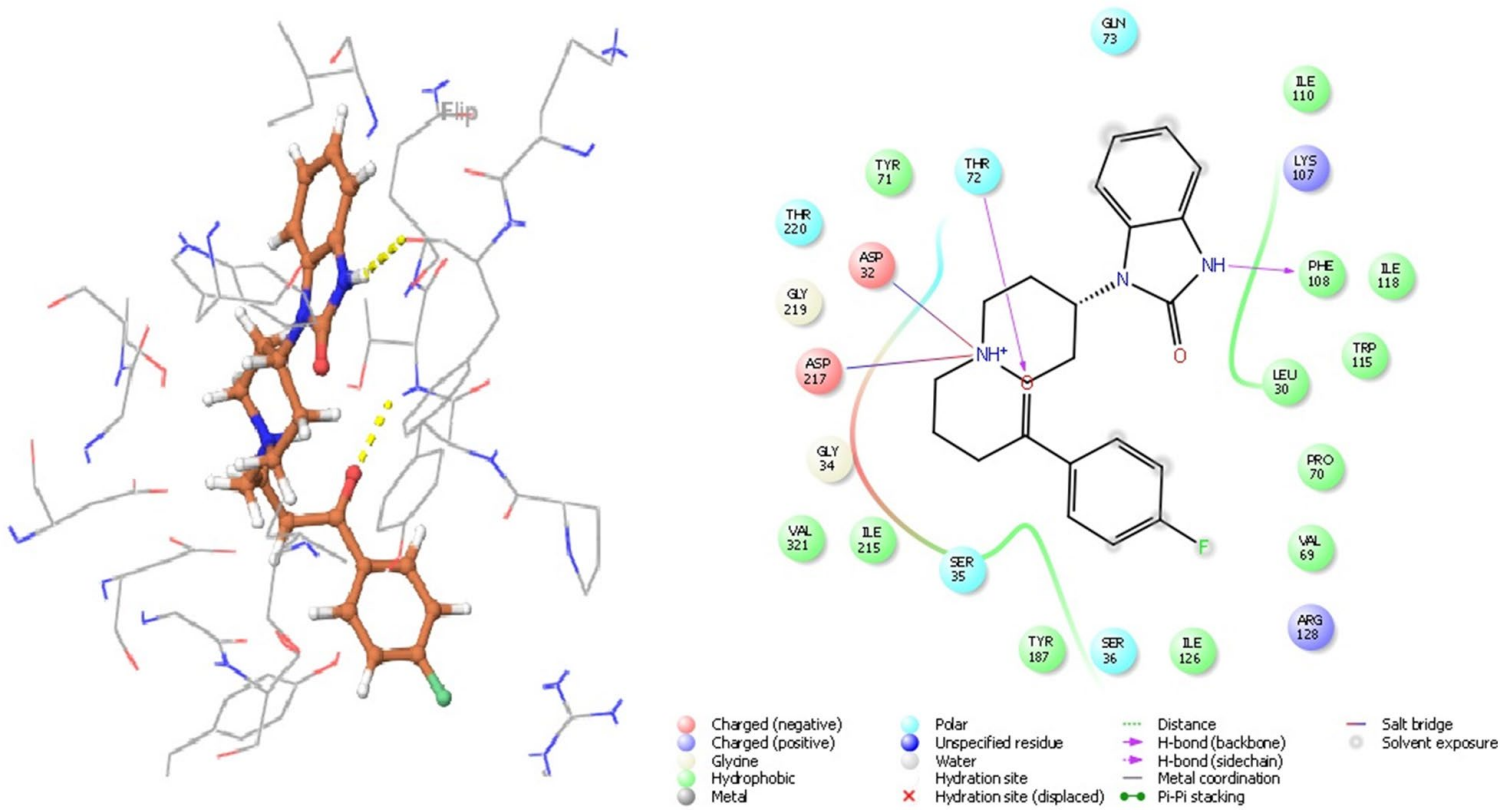
The lowest energy conformation of docking result of Benperidol with BACE 1 (4D8C)

Ligand interaction in BACE 1 depends on the active site residues conformation, which consisted of catalytic dyad (Asp32, Asp228), 10 s loop composed of residues 9–14, flap consisting of amino acids 67–77 and all other residues within 8 Å from aspartates. The flap occurs in three form close form, close to open form and transition form. Therefore, to avoid biasness we chose two BACE 1 complex, 3L5E (transition form) and 4D8C (open form) [19]. With BACE 1 (4D8C), benperidol showed that highest docking score of − 6.854 and glide energy of − 54.082 kcal/mol, known inhibitor LY2886721 had docking score of − 6.80 and glide energy of − 35.835 kcal/mol. In this case, hydrogen bonding was observed with Thr72 and Phe108 whereas salt bridge with Asp32 and Asp217 (catalytic residues) (Tables 1, 2 and Fig. 6). Anisoperidone was best docked with BACE 1 (3l5e) with a score of − 8.513 and glide energy of − 43.063 kcal/mol whereas the known inhibitor LY2886721 had dock score of − 6.123 and glide energy of − 35.835 kcal/mol. It forms hydrogen binding with Trp137, Trp259; pi-cation with Tyr132 and salt bridge with Asp93 (Tables 1, 2 and Fig. 7).

### Molecular interaction of the anisopirol with *N*-methyl-d-aspartate receptor

**Fig. 8:**
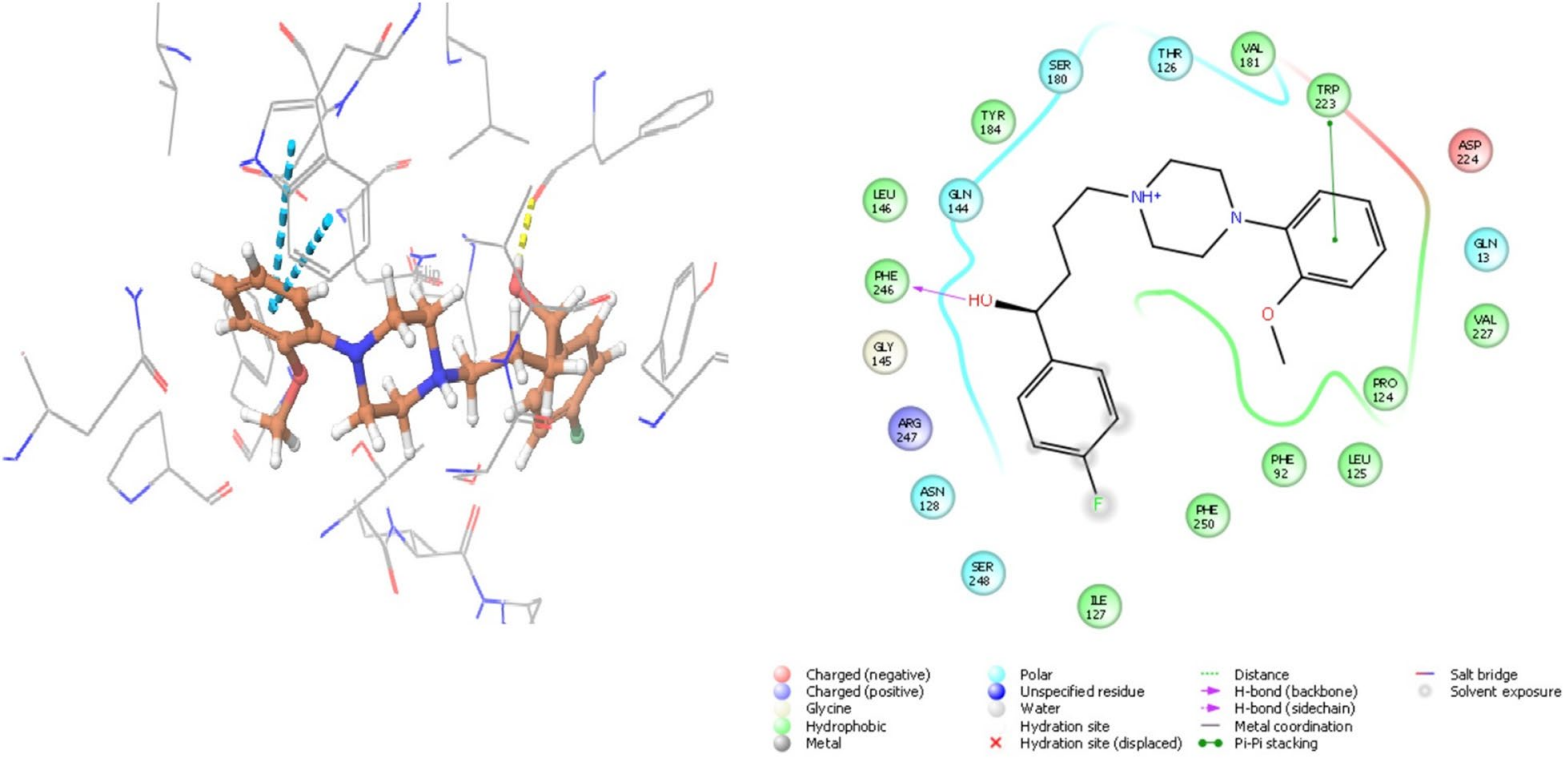
The lowest energy conformation of docking result of Anisopirol with NMDA (1PBQ)

For protein 1PBQ, crucial interacting residues with co crystallised ligand DCKA are Pro124, Ser180, Thr126 and Arg131. Hydrophobic pocket of 1PBQ has following amino acid residues: Phe16, Phe92, Trp223 and Phe250 [50]. Anisopirol showed the best docking score of − 6.903 and glide energy of − 36.533 kcal/mol whereas, DCKA showed a docking score of − 14.084 and glide energy of − 43.865 kcal/mol. Hydrogen bonding was observed between anisopirol and Phe246 whereas pi–pi stacking with Trp223, polar interactions with Thr126 and Ser180 whereas hydrophobic interactions are observed with Pro124, Phe92 of 1PBQ (Tables 1, 2 and Fig. 8).

### Prediction of pharmacokinetic properties

Pharmacokinetics depends upon the molecular descriptors of the drug candidate. In silico prediction of absorption, distribution metabolism and excretion (ADME) properties have become important in drug selection and to determine its success for human therapeutic use. Therefore, these physio-chemical descriptors were calculated so as to determine the ADME properties of the drugs. Lipinski’s rule of five is based on the observation that drugs with molecular weight of 500 Da or less, donorHB ≤ 5, accptHB ≤ 10 and QPlogPo/w ≤ 5 are orally administered drugs. Molecules which are not following more than one of these four rules may have problem with bioavailability. None of the drugs in present study violated the Lipinski’s rule of five. Percentage human oral absorption was calculated to predict the oral absorption on the scale of on 0–100%. More than 80% are thought to have as high absorption whereas any compound with less than 25% is poor. According to this criterion; all the drugs have medium to high oral absorption ranging from 53.364 to 100% (Table 3).

**Table 3:** Molecular descriptor values of the top interacting antipsychotic drugs

S. no	Name of the drug	Molecular weight	QPlogPo/w	DonorHB	accptHB	Percent human-oral absorption	Violation of Lipinski’s rule
1	Sulmepride	242.031	1.46	1	2	82.403	0
2	Promazine hydrochloride	214.221	− 0.867	4	6.9	53.364	0
3	Bromperidol	223.069	4.368	1	4.75	100	0
4	Anisopirol	358.455	4.047	1	5.45	100	0
5	Melperone	263.354	2.944	0	4	96.488	0
6	Pimozide	461.555	6.413	1	4	96.301	1
7	Benperidol	381.449	3.45	1	6	86.914	0
8	Azabuperone	290.38	1.905	0	6	79.665	0
9	Anisoperidone	335.445	4.126	0	4.75	100	0

## Discussion

Over the past years, de novo drug discovery has faced some serious issues due to its cost and time consumption. While the investment has increased in the pharmaceutical companies, the number of new approved drugs has been stationary; therefore, in silico drug repurposing is an efficient and encouraging tool for discovering new uses from already existing drugs. There are various examples of repurposed drugs which were discovered by computational approach and are now being used in other diseases. For example, Raltegravir, originally an HIV-1 integrase inhibitor is now used as adjuvant therapy in cancer [51] and Valsartan, an angiotensin receptor blocker is now being used for AD [52]. Complex pathophysiology of AD suggests multi-target strategies and drugs with polypharmacological interactions. In the present study, we are focussing on the conventional (AChE, BuChE and NMDA) as well as new experimental (BACE 1, MAO A) targets of AD. For each receptor, about three to five drug poses were analysed to identify the pose with least docking score and the minimum binding energy. The docking score of top nine interacting antipsychotic drugs with all the target protein (AChE, BuChE, BACE 1, MAO A, and NMDA) were studied and their pharmacologically active protein target was also predicted (Tables 1 and 4).

**Table 4:** Top interacting drug with its pharmacological known and predicted protein target

Drug name	IUPAC name	Drug structure	Known pharmacological target	Predicted drug target
Pimozide	3-[1-[4,4-bis(4-fluorophenyl)butyl]piperidin-4-yl]-1H-benzimidazol-2-one		Dopamine D2 receptor [53]	AChE
Bromperidol	4-[4-(4-bromophenyl)-4-hydroxypiperidin-1-yl]-1-(4-fluorophenyl)butan-1-one		Dopamine D2 receptor [54]	BuChE
Melperone	1-(4-fluorophenyl)-4-(4-methylpiperidin-1-yl)butan-1-one		Dopamine D2/D3 and 5-HT_2A_ antagonist [55]	MAO A
Anisoperidone	1-(4-methoxyphenyl)-4-(4-phenyl-3,6-dihydro-2H-pyridin-1-yl)butan-1-one		Dopamine D2 and 5-HT2A receptors [56]	BACE 1 (3L5E)
Benperidol	3-[1-[4-(4-fluorophenyl)-4-oxobutyl]piperidin-4-yl]-1H-benzimidazol-2-one		Dopamine D2 antagonist [57]	BACE 1 (4D8C)
Anisopirol	1-(4-fluorophenyl)-4-[4-(2-methoxyphenyl)piperazin-1-yl]butan-1-ol		Dopamine receptor [58]	NMDA

**Fig. 9:**
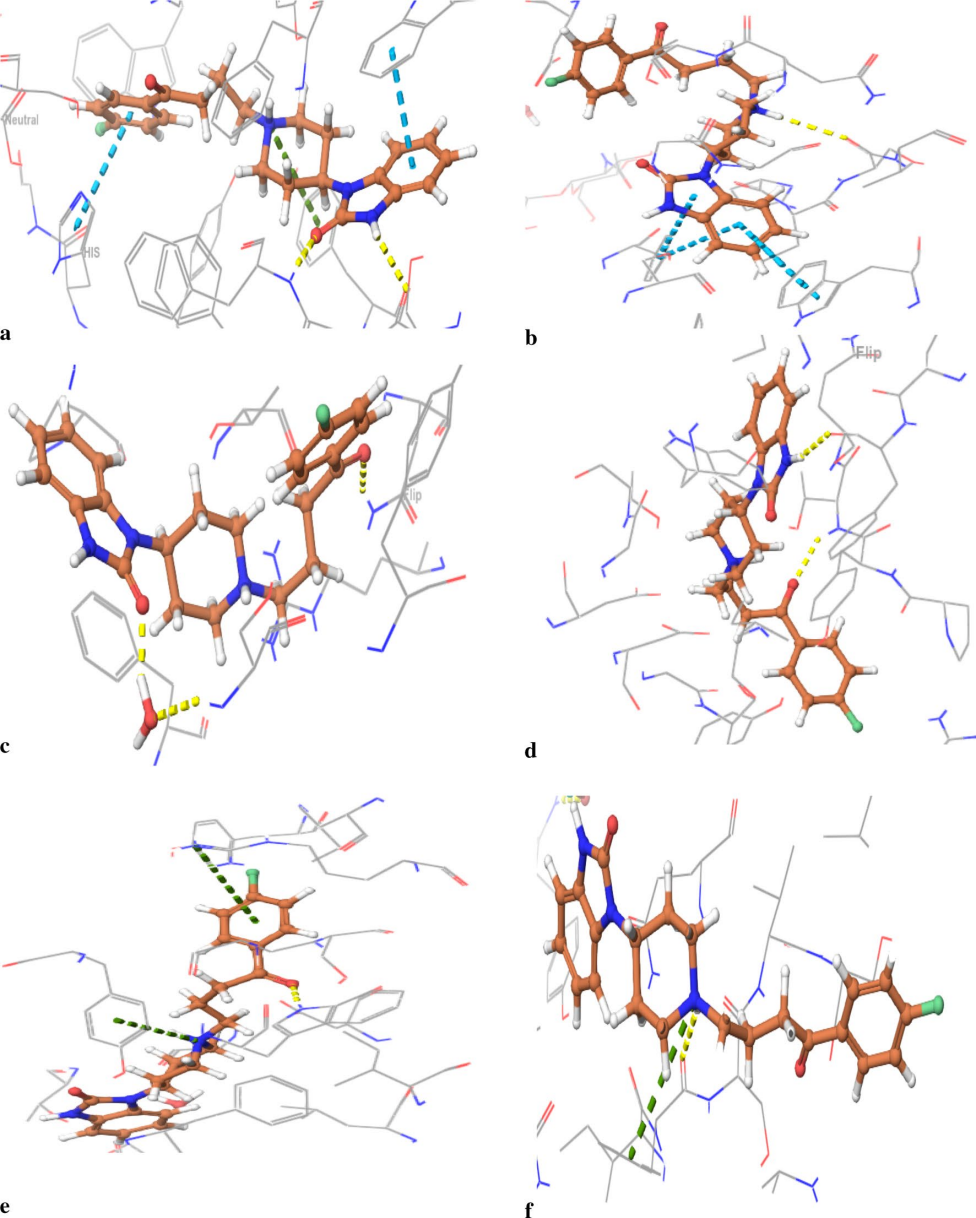
Molecular interaction of the Benperidol with target proteins (**a** 4EY6; **b** 1P0M; **c**1PBQ; **d** 4D8C; **e** 3L5E; **f** 2Z5X)

The other drugs were also found to be display good molecular interaction with certain protein. For example, Pimozide showed best interaction with AChE, Bromperidol with BuChE, Melperone with MAO A, Anisopirol with NMDA, Anisoperidone with BACE 1 (3L5E) and Benperidol with BACE 1 (4D8C). In accordance to this approach, Benperidol, a butyrophenone derivative, was found to be the best candidate drug based on its dock score (Table 1), glide energy and molecular interactions with all target protein (Table 5 and Fig. 9). The second best candidate, Melperone was of same class of compound as that of Benperidol, suggesting potent role of their basic skeletal structure in the interaction with the target proteins. For proteins MAO A and BACE 1, Melperone showed lower docking score as compared to Benperidol. This observation suggests that further modification in their basic structure and formation of some new analogs may enhance their multi potent anti-Alzheimer’s activity.

**Table 5:** Molecular interaction of Benperidol with different protein targets

AChE (4EY6)		BuChE (1P0M)		MAO A (2Z5X)		BACE 1 (4D8C)		BACE 1 (3L5E)		NMDA (1PBQ)	
Glide energy	Interacting residue	Glide energy	Interacting residue	Glide energy	Interacting residue	Glide energy	Interacting residue	Glide energy	Interacting residue	Glide energy	Interacting residue
− 46.208	Trp286 (π–π stacking) Tyr337 (π–π stacking) His447 (π–π stacking) Ser293 (H bond) Phe295 (H bond)	− 44.437	Ser287 (H bond) Phe329 (π–π stacking) Trp231 (π–π stacking)	− 54.432	Phe208 (H bond) Phe208 (π-cation)	− 54.082	Thr72 (H bond) Phe108 (H bond) Asp32 (salt bridge) Asp217 (salt bridge)	− 48.264	Trp137 (H bond) Tyr132 (π-cation) Arg189 (π-cation) Asp93 (salt bridge)	− 37.114	Asn128 (H bond)

A lead molecule is considered to be a successful oral drug when it is quickly and completely absorbed from the gastrointestinal tract, distributed to the specific site in the body where it has to act, metabolised in such a way that it doesn’t immediately abolish its activity and should eliminate from the body without causing any harm to any organ. Because of poor pharmacokinetics, half of the developed drugs fail to reach markets. Benperidol and Melperone didn’t violated Lipinski’s rule of five and showed 86.914 and 96.488% human oral absorption.

With in silico repurposing approach, there can be a possibility of false positive hits during screening and also the activity of the candidate drug molecules may vary in the in vitro or in vivo systems. Therefore, to validate their potency further in vitro and in vivo studies are needed to be performed.

## Conclusion

AD is a complex neurodegenerative disease involving multiple targets such as AChE, BuChE, BACE 1, MAO A and NMDA. Several molecules have been developed against these targets to alleviate the symptoms and having disease modifying effects. In spite of the laborious efforts, presently very few drugs are in the pipeline due to the limitation associated with molecule to satisfy ADME profile. Due to these limitations the present study explored the repurposing of already known antipsychotic drugs, which means their pharmacokinetics, toxicology profile, formulation development and bulk manufacturing have already been done hence saving cost and time. Of all the antipsychotic drugs studied, Benperidol was found to be the best candidate for the cholinergic (AChE and BuChE), monoaminergic (MAO A), glutamatergic system (NMDA) and beta-secretase cleavage enzyme (BACE 1). Hence, in silico drug repurposing has been able to identify promising results which might be useful therapeutically in AD.

## Abbreviations

AD: Alzheimer’s disease
AChE: acetylcholinesterase
CNS: central nervous system
ACh: acetylcholine
BuChE: butyrylcholinesterase
ChAT: choline acetyltransferase
PAS: peripheral anionic site
BACE 1: beta secretase cleavage enzyme 1
γ-Secretase: gamma secretase
Aβ: β-amyloid
APP: amyloid precursor protein
ROS: reactive oxygen species
MAO: monoamine oxidase
NMDA: *N*-methyl-d-aspartate
Ca^2+^: calcium ions
FDA: Food and Drug Administration, USA
BPSD: behavioural and psychological symptoms of dementia
5-HT: 5-hydroxytryptamine receptors
PDB: Protein Data Bank
OPLS: optimized potentials for liquid simulations
Glide: grid based ligand docking with energetics
CPU: Central Processing Unit
RAM: Random Access Memory
MW: molecular weight
QPlogPo/w: predicted octanol/water partition coefficient
donorHB: number of hydrogen bond donors
acceltHB: number of hydrogen bond acceptors
RMSD: root mean square deviation
ΔG_ecol_: energy contributed due to electrostatic bonding
ΔG_edw_: energy contributed due to Van der Waals bonding
Xphbond: energy contributed due to hydrogen bonding
ADME: absorption, distribution, metabolism and excretion

## References

[1] Cui Z, Sheng Z, Yan X, Cao Z, Tang K (2016). *In Silico* Insight into Potential Anti-Alzheimer’s Disease Mechanisms of Icariin. Int J Mol Sci.

[2] Mantoani SP, Chierrito TP, Vilela AF, Cardoso CL, Martínez A, Carvalho I (2016). Novel triazole-quinoline derivatives as selective dual binding site acetylcholinesterase inhibitors. Molecules.

[3] Alzheimer’s A (2015). Alzheimer’s disease facts and figures. Alzheimers Dement.

[4] Shaik AS, Raja AE, Vijayalakshmi M, Devalarao G (2010). Alzheimer’s disease: pathophysiology and treatment. Int J Pharm Biosci.

[5] Kim TW (2015). Drug repositioning approaches for the discovery of new therapeutics for Alzheimer’s disease. Neurotherapeutics.

[6] Rochais C, Lecoutey C, Gaven F, Giannoni P, Hamidouche K, Hedou D, Dubost E, Genest D, Yahiaoui S, Freret T, Bouet V (2015). Novel multitarget-directed ligands (MTDLs) with acetylcholinesterase (AChE) inhibitory and serotonergic subtype 4 receptor (5-HT4R) agonist activities as potential agents against Alzheimer’s disease: the design of donecopride. J Med Chem.

[7] Corbett A, Pickett J, Burns A, Corcoran J, Dunnett SB, Edison P, Hagan JJ, Holmes C, Jones E, Katona C, Kearns I (2012). Drug repositioning for Alzheimer’s disease. Nat Rev Drug Discov.

[8] Nikolic K, Mavridis L, Djikic T, Vucicevic J, Agbaba D, Yelekci K, Mitchell JB (2016). Drug design for CNS diseases: polypharmacological profiling of compounds using cheminformatic, 3D-QSAR and virtual screening methodologies. Front Neurosci.

[9] Mucke HA (2015). The case of galantamine: repurposing and late blooming of a cholinergic drug. Future Sci OA.

[10] Oprea TI, Mestres J (2012). Drug repurposing: far beyond new targets for old drugs. AAPS J.

[11] Corbett A, Williams G, Ballard C (2015). Drug repositioning in Alzheimer’s disease. Front Biosci (Schol Ed).

[12] Nelson PT, Alafuzoff I, Bigio EH, Bouras C, Braak H, Cairns NJ, Castellani RJ, Crain BJ, Davies P, Del Tredici K, Duyckaerts C (2012). Correlation of Alzheimer disease neuropathologic changes with cognitive status: a review of the literature. J Neuropathol Exp Neurol.

[13] Jackson-Siegal J (2005). Our current understanding of the pathophysiology of Alzheimer’s disease. Adv Stud Pharm.

[14] Kumar S, Chowdhury S (2014). Kinetics of acetylcholinesterase inhibition by an aqueous extract of *Cuminum cyminum* seeds. Int J Appl Sci Biotechnol.

[15] Kumar S, Chowdhury S (2015). Kinetics of inhibition of butyrylcholinesterase by an aqueous extract of *Cuminum cyminum*. Pharm Biol Eval.

[16] Ul-Haq Z, Khan W, Kalsoom S, Ansari FL (2010). *In silico* modeling of the specific inhibitory potential of thiophene-2, 3-dihydro-1,5-benzothiazepine against BChE in the formation of β-amyloid plaques associated with Alzheimer’s disease. Theor Biol Med Model.

[17] Dickerson TJ, Beuscher AE, Rogers CJ, Hixon MS, Yamamoto N, Xu Y, Olson AJ, Janda KD (2005). Discovery of acetylcholinesterase peripheral anionic site ligands through computational refinement of a directed library. Biochemistry.

[18] Zhang YW, Thompson R, Zhang H, Xu H (2011). APP processing in Alzheimer’s disease. Mol Brain.

[19] Hilpert H, Guba W, Woltering TJ, Wostl W, Pinard E, Mauser H, Mayweg AV, Rogers-Evans M, Humm R, Krummenacher D, Muser T (2013). β-Secretase (BACE1) inhibitors with high in vivo efficacy suitable for clinical evaluation in Alzheimer’s disease. J Med Chem.

[20] Cole SL, Vassar R (2008). The role of amyloid precursor protein processing by BACE1, the β-secretase. Alzheimer disease pathophysiology. J Biol Chem.

[21] Aprahamian I, Stella F, Forlenza OV (2013). New treatment strategies for Alzheimer’s disease: is there a hope?. Indian J Med Res.

[22] Chauhan V, Chauhan A (2006). Oxidative stress in Alzheimer’s disease. Pathophysiology.

[23] Huang WJ, Zhang X, Chen WW (2016). Role of oxidative stress in Alzheimer’s disease. Biomed Rep.

[24] Son SY, Ma J, Kondou Y, Yoshimura M, Yamashita E, Tsukihara T (2008). Structure of human monoamine oxidase A at 2.2-Å resolution: the control of opening the entry for substrates/inhibitors. Proc Natl Acad Sci USA.

[25] Binda C, Wang J, Pisani L, Caccia C, Carotti A, Salvati P, Edmondson DE, Mattevi A (2007). Structures of human monoamine oxidase B complexes with selective noncovalent inhibitors: safinamide and coumarin analogs. J Med Chem.

[26] Veitinger M, Varga B, Guterres SB, Zellner M (2014). Platelets, a reliable source for peripheral Alzheimer’s disease biomarkers?. Acta Neuropathol Commun.

[27] Finberg JP, Rabey JM (2016). Inhibitors of MAO-A and MAO-B in psychiatry and neurology. Front Pharmacol.

[28] Esposito Z, Belli L, Toniolo S, Sancesario G, Bianconi C, Martorana A (2013). Amyloid β, glutamate, excitotoxicity in Alzheimer’s disease: are we on the right track?. CNS Neurosci Ther.

[29] Danysz W, Parsons CG (2012). Alzheimer’s disease, β-amyloid, glutamate, NMDA receptors and memantine–searching for the connections. Br J Pharmacol.

[30] Butterfield DA, Pocernich CB (2003). The glutamatergic system and Alzheimer’s disease. CNS Drugs.

[31] Mitra A, Dey B. Therapeutic interventions in Alzheimer disease (Chap. 12). In: Kishore U, editor Neurodegenerative diseases. Intech; 2013. p. 291–317. doi:10.5772/54915.

[32] Parsons CG, Danysz W, Dekundy A, Pulte I (2013). Memantine and cholinesterase inhibitors: complementary mechanisms in the treatment of Alzheimer’s disease. Neurotox Res.

[33] Cerejeira J, Lagarto L, Mukaetova-Ladinska E (2012). Behavioral and psychological symptoms of dementia. Front Neurol.

[34] Parida PR, Yadav RN, Shankar BR, Chakraborty DI, Das A, Singh NK (2012). *In*-*silico* protein ligand interaction study of typical antipsychotic drugs against dopaminergic D2 receptor. Int J Pharm Pharmceusci.

[35] Meltzer HY (1999). The role of serotonin in antipsychotic drug action. Neuropsychopharmacology.

[36] Filip M, Bader M (2009). Overview on 5-HT receptors and their role in physiology and pathology of the central nervous system. Pharmacol Rep.

[37] Chowdhury S, Kumar S (2016). *In vitro* anti-acetylcholinesterase activity of an aqueous extract of *Unicaria tomentosa* and in silico study of its active constituents. Bioinformation.

[38] Nicolet Y, Lockridge O, Masson P, Fontecilla-Camps JC, Nachon F (2003). Crystal structure of human butyrylcholinesterase and of its complexes with substrate and products. J Biol Chem.

[39] Bajda M, Jończyk J, Malawska B, Filipek S (2014). Application of computational methods for the design of BACE-1 inhibitors: validation of in silico modelling. Int J Mol Sci.

[40] Krueger BA, Weil T, Schneider G (2009). Comparative virtual screening and novelty detection for NMDA-GlycineB antagonists. J Comput Aided Mol Des.

[41] Turkmenoglu FP, Baysal İ, Ciftci-Yabanoglu S, Yelekci K, Temel H, Paşa S, Ezer N, Çalış İ, Ucar G (2015). Flavonoids from Sideritis species: human monoamine oxidase (hMAO) inhibitory activities, molecular docking studies and crystal structure of xanthomicrol. Molecules.

[42] Sastry GM, Adzhigirey M, Day T, Annabhimoju R, Sherman W (2013). Protein and ligand preparation: parameters, protocols, and influence on virtual screening enrichments. J Comput Aided Mol Des.

[43] Schrödinger Release 2015-4: LigPrep, version 3.6, Schrödinger, LLC, New York, 2015.

[44] Friesner RA, Murphy RB, Repasky MP, Frye LL, Greenwood JR, Halgren TA, Sanschagrin PC, Mainz DT (2006). Extra precision glide: docking and scoring incorporating a model of hydrophobic enclosure for protein-ligand complexes. J Med Chem.

[45] Small-Molecule Drug Discovery Suite 2015-4: QikProp, version 4.6, Schrödinger, LLC, New York, 2015.

[46] Wang R, Lu Y, Wang S (2003). Comparative evaluation of 11 scoring functions for molecular docking. J Med Chem.

[47] Johnson G, Moore SW (2006). The peripheral anionic site of acetylcholinesterase: structure, functions and potential role in rational drug design. Curr Pharm Des.

[48] Çokuğraş AN (2003). Butyrylcholinesterase: structure and physiological importance. Turk J Biochem.

[49] Edmondson DE, Binda C, Wang J, Upadhyay AK, Mattevi A (2009). Molecular and mechanistic properties of the membrane-bound mitochondrial monoamine oxidases. Biochemistry.

[50] Sharma M, Gupta VB (2012). Dual allosteric effect in glycine/NMDA receptor antagonism: a molecular docking simulation approach. Int J Drug Des Discov.

[51] Oprea TI, Bauman JE, Bologa CG, Buranda T, Chigaev A, Edwards BS, Jarvik JW, Gresham HD, Haynes MK, Hjelle B, Hromas R (2012). Drug repurposing from an academic perspective. Drug Discov Today Ther Strateg.

[52] Lee HM, Kim Y (2016). Drug repurposing is a new opportunity for developing drugs against neuropsychiatric disorders. Schizophr Res Treat.

[53] Seeman P, Lee T (1975). Antipsychotic drugs: direct correlation between clinical potency and presynaptic action on dopamine neurons. Science.

[54] Suzuki A, Kondo T, Mihara K, Yasui-Furukori N, Otani K, Furukori H, Kaneko S, Inoue Y (2001). Association between TaqI A dopamine D2 receptor polymorphism and therapeutic response to bromperidol: a preliminary report. Eur Arch Psychiatry Clin Neurosci.

[55] Meltzer HY, Matsubara S, Lee JC (1989). Classification of typical and atypical antipsychotic drugs on the basis of dopamine D-1, D-2 and serotonin2 pKi values. J Pharm Exp Ther.

[56] Horacek J, Bubenikova-Valesova V, Kopecek M, Palenicek T, Dockery C, Mohr P, Höschl C (2006). Mechanism of action of atypical antipsychotic drugs and the neurobiology of schizophrenia. CNS Drugs.

[57] Moerlein SM, Perlmutter JS, Markham J, Welch MJ (1997). *In vivo* kinetics of [18F](*N*-methyl) benperidol: a novel PET tracer for assessment of dopaminergic D2-like receptor binding. J Cereb Blood Flow Metab.

[58] Fielding S, Lal H (1978). Behavioral actions of neuroleptics. Handb Psychopharmacol.

